# MicroRNA-155 Controls T Helper Cell Activation During Viral Infection

**DOI:** 10.3389/fimmu.2019.01367

**Published:** 2019-06-13

**Authors:** Eliana Goncalves-Alves, Victoria Saferding, Christopher Schliehe, Robert Benson, Mariola Kurowska-Stolarska, Julia Stefanie Brunner, Antonia Puchner, Bruno K. Podesser, Josef S. Smolen, Kurt Redlich, Michael Bonelli, James Brewer, Andreas Bergthaler, Günter Steiner, Stephan Blüml

**Affiliations:** ^1^Department of Rheumatology, Medical University Vienna, Vienna, Austria; ^2^Ludwig Boltzmann Institute for Arthritis and Rehabilitation, Vienna, Austria; ^3^CeMM - Center for Molecular Medicine of the Austrian Academy of Sciences, Vienna, Austria; ^4^Institute of Infection, Immunity and Inflammation, University of Glasgow, Glasgow, United Kingdom; ^5^Institute for Vascular Biology and Thrombosis Research, Medical University of Vienna, Vienna, Austria; ^6^Christian Doppler Laboratory for Arginine Metabolism in Rheumatoid Arthritis and Multiple Sclerosis, Vienna, Austria; ^7^Department of Biomedical Research, Medical University of Vienna, Vienna, Austria

**Keywords:** microRNA-155, antiviral immunity, T helper cells, T cell activation, APCs, APC-T cell interaction

## Abstract

MicroRNA (miR) 155 has been implicated in the regulation of innate and adaptive immunity as well as autoimmune processes. Importantly, it has been shown to regulate several antiviral responses, but its contribution to the immune response against cytopathic viruses such as vesicular stomatitis virus (VSV) infections is not known. Using transgenic/recombinant VSV expressing ovalbumin, we show that miR-155 is crucially involved in regulating the T helper cell response against this virus. Our experiments indicate that miR-155 in CD4^+^ T cells controls their activation, proliferation, and cytokine production *in vitro* and *in vivo* upon immunization with OVA as well as during VSV viral infection. Using intravital multiphoton microscopy we analyzed the interaction of antigen presenting cells (APCs) and T cells after OVA immunization and found impaired complex formation when using miR-155 deficient CD4^+^ T cells compared to wildtype CD4^+^ T cells *ex vivo*. In contrast, miR-155 was dispensable for the maturation of myeloid APCs and for their T cell stimulatory capacity. Our data provide the first evidence that miR-155 is required for efficient CD4^+^ T cell activation during anti-viral defense by allowing robust APC-T cell interaction required for activation and cytokine production of virus specific T cells.

## Introduction

T cell activation has to be tightly controlled to allow an efficient immune response against infectious diseases but also to prevent autoimmunity. MicroRNAs (miRs) have been implicated in both processes and are therefore interesting targets to study immune regulation. In particular, miR-155 has been shown to regulate many aspects of the immune system (1–3). It is involved in the regulation of macrophage and dendritic cell biology, including cytokine production by targeting PU-1 and SHIP-1 (4–6). In B cells, this microRNA has been demonstrated to control germinal center formation and the generation of class switched antibodies (7–9). CD4^+^ T cells of miR-155 deficient mice were shown to have a bias toward a Th2 phenotype, while overexpression of miR-155 promotes a Th1 phenotype (7, 10). The increased Th2 differentiation potential of miR-155 deficient CD4^+^ T cells was attributed to an up-regulation of c-Maf, a potent transcription factor of the IL-4 promoter and a miR-155 target (7). MiR-155 also targets IFNγRα in CD4^+^ cells cultured under Th1 conditions, which is one of the multiple known targets responsible for the inhibition of Th1 differentiation of miR-155^−/−^ CD4^+^ T helper cells (10). MiR-155 deficient mice have an impaired capacity for polarization of T helper cells into Th17 and follicular T helper cells (11, 12).

We and others have previously shown, that miR-155 deficient mice are protected from collagen induced arthritis due to reduced generation of autoreactive T and B cells (13, 14). Also, in experimental autoimmune encephalitis (EAE), protection from disease in miR-155 deficient mice was mediated primarily by reduced generation of pathogenic T cells. In arthritis, miR-155 also plays an important role in myeloid cells, since it controls the expression of pro-inflammatory cytokines and chemokines in monocytes and macrophages (14). In addition, miR-155 has been implicated in dendritic cell (DC) function, as it is highly upregulated during their maturation. However, its functional role in DCs has remained controversial (5, 6, 15–17).

During host response to viral infections, miR-155 has been demonstrated to be critically involved in CD8^+^ cytotoxic T cell activation by controlling interferon signaling and SOCS1 expression (18). This mechanism has been shown to be very important in host response to viral infections, especially in lymphocytic choriomeningitis virus (LCMV) and influenza infection (18, 19). In macrophages infected with vesicular stomatitis virus (VSV), miR-155 was shown to be important to control viral replication via targeting SOCS1 *in vitro* (20).

Cytopathic viruses such as VSV and vaccinia do not essentially require CD8^+^ T cells for host defense, but crucially rely on CD4^+^ T helper cells and neutralizing antibody producing B cells (21–25). However, the role of miR-155 in this process is not known. We have therefore analyzed the role of miR-155 in T helper cell responses toward vesicular stomatitis virus (VSV) using recombinant viruses expressing ovalbumin, which allowed us to track antiviral T cell responses using ovalbumin-specific T cell receptor (TCR) transgenic OTII T cells.

## Materials and Methods

### Animals

All mice used were on a C57BL/6 background. MiR-155^−/−^, wild-type (WT), ovalbumin-specific Tcrα/Tcrβ transgenic (OTII) mice were obtained from Jackson Laboratories and bred in house (Biomedical Research Facility, Medical University of Vienna). To obtain miR-155^−/−^ OTII mice and miR-155^+/+^ OTII littermates, WT OTII mice were crossed with miR-155^−/−^ mice. CD45.1 mice were kindly provided by the group of Dr. Silvia Knapp (Medical University of Vienna), transgenic mice carrying the IghelMD4 transgene that recognizes hen egg lysozyme (HEL) and CD11c-YFP mice were bred in the Center Research, University of Glasgow. All animals, except the CD45.1 mice, express the *Ptpr*c^*b*^ (CD45.2) allele. All animal studies were approved by the animal ethics committee from the Medical University Vienna and the University of Glasgow and comply with institutional guidelines.

### Preparation of Primary Cells, Mixed Lymphocyte Reaction, Proliferation Assays

Dendritic cells (DCs) were generated from WT or miR-155^−/−^ mice similarly as described before (Lutz et al). Briefly, bone marrow cells flushed from femur and tibia of mice were cultured in complete RPMI-1640 medium containing 10% fetal bovine serum, 2 mM L-glutamine, penincilin (100 U/mL), streptomycin (100 ug/mL) (all from Gibco) supplemented with 20 ng/mL mGM-CSF (R&D). After 7–9 days of culture, BMDCs were matured for 24 h in complete RPMI supplemented with 10 μg/mL *E.coli* LPS (Sigma). For mixed lymphocyte reaction (MLR), CD4^+^ cells were isolated from splenocytes of WT or miR-155^−/−^ OTII mice by magnetic cell separation (MACS) using the CD4^+^ isolation kit according to manufacture recommendations (Miltenyi Biotec, Germany), and co-cultured with DCs in the presence of Ovalbumin (OVA) or ovalbumin peptide 323–339 (pOVA) (both AnaSpec, CA, USA) in the indicated concentrations for 96 h. Alternatively, MACS isolated CD4^+^ splenocytes were cultured in complete RPMI in 96 well plates (100,000 cells/well) coated with anti-CD28 (3 μg/mL) and anti-CD3 (1 μg/mL; both from BioXCell, NH, USA) for 96 h. Cell proliferation was quantified by H^3^-Thymidine (1 μCi/well) incorporation during the last 18 h of culture and presented as mean value of six technical replicates per condition, both in the MLR and in the plate bound antibody-induced proliferation assays. Quantification was done on a TopCount^®^NXTTM microplate scintillation counter (Packard, CT, USA).

### Adoptive Cell Transfer

Magnetically separated WT or miR-155^−/−^ OTII CD4^+^ T cells, were labeled with 10 μg/mL CFSE (life technologies, CA, USA) in PBS for 20 min at 37°C as recommended by the manufacturer (10 × 10^6^ cells/ml). After labeling, 2–5 × 10^6^ cells/mouse were injected intravenously into either WT, miR-155^−/−^ or CD45.1 mice. For multiphoton imaging, 2–5 × 10^6^ cells/mouse were labeled with 5 μM Cell Tracker Red (CMTPX, Invitrogen, Paisley, Scotland, UK) in CO_2_-independent media (Invitrogen, Paisley, Scotland, UK) for 40 min at 37°C, and injected *i.v*. into CD11c-YFP mice. For immunization experiments with HEL-OVA, OTII T cells, and MD4 B cells were prepared and injected as described before (26).

### RVSV-OVA Infection and Protein Immunization

Recombinant VSV-OVA virus (rVSV-OVA) was kindly provided by Mike Bevan and was initially designed by Dr. Leo LeFrancois (27). For the rVSV-OVA infection each WT or miR-155^−/−^ mice received intravenously 2 × 10^5^ plaque forming units (PFU) of rVSV-OVA diluted in BSS. CD45.1 mice were infected with the same amount of virus 1 day after adoptive T cell transfer. Eight days after infection, blood and spleen were collected from each animal. Protein immunizations after adoptive T cell transfer were done either *i.v*. with a solution of LPS and OVA (75 and 30 μg/mouse, respectively) or *s.c*. in the footpad with 130 μg conjugated HEL-OVA in complete Freund's adjuvant (Sigma), prepared as previously described (28). In mice immunized with LPS and OVA spleens were collected 96 h after immunization; in mice immunized with HEL-OVA the axial and brachial LNs were collected 6 days after immunization.

### *In vitro* Re-stimulation Assay

Splenocytes (1 × 10^5^ per well) were cultured in complete RPMI in the presence of 5 μg/mL of pOVA (ovalbumin peptide 323–339, AnaSpec) in a 96 well plate for 96 h, 50 μL of cell culture supernatant were collected for cytokine quantification. Cell proliferation was quantified by H^3^-Thymidine (1 μCi/well) incorporation in the last 18 h of culture, six technical replicates were used per mouse. Quantification was on a TopCount^®^NXT^TM^ microplate scintillation counter (Packard, CT, USA).

### Neutralizing Antibody Assay

VSV-neutralizing antibody titers were quantified as previously described (29). Concisely, mouse serum was diluted 40-fold in MEM containing 5% FCS and heat inactivated for 30 min at 56°C. Serial 2-fold dilutions were incubated for 90 min at 37°C in an equal volume of rVSV-OVA solution containing 1,500 PFU/mL. Serum-virus solution was transferred onto Vero cell (ATCC, VI, USA) monolayers and incubated for 90 min at 37°C, after which the cells were overlaid with MEM containing 1% methylcellulose. Cells were fixed and stained with 0.5% crystal violet solution after 24 h of incubation at 37°C, 5% CO_2_. Antibody titer was calculated as the highest dilution of serum that reduced the number of plaques by at least 50%.

### Antibodies ELISA and FlowCytomix Analysis

Anti-OVA and Anti-HEL antibodies were detected as previously described (28). Soluble forms of IL-2, TNFα, IFN-γ, and IL-4 were detected using the FlowCytomix kit according to the manufacturer's recommendations (Bender MedSystems, GmbH, Vienna, Austria).

### Real Time PCR

RNA isolation of sorted dendritic cells and stimulated T cells was done using the RNeasy Mini kit (Qiagen), for cDNA synthesis the Omniscrip kit (Qiagen) was used. Real-time PCR was done in a Light cycler 480 (Roche) using SYBR^®^ green master mix and the following primer pairs: IL-12p40 fwd: 5′-GAC ACG CCT GAA GAA GAT GAC-3′, rev.: 5′-TAG TCC CTT TGG TCC AGT GTG-3′; IL-12p35 fwd: 5′-CCC TTG CCC TCC TAA ACC AC-3′, rev: 5′-AAG GAA CCC TTA GAG TGC TTA CT-3′; IL-23 fwd: 5′-ATG CTG GAT TGC AGA GCA GTA-3′ rev: 5′-ACG GGG CAC ATT ATT TTT AGT CT-3′; IL-6 fwd: 5′- GCC CAA ACA CCA AGT CAA GT-3′ rev: 5′-TAT AGG AAA CAG CGG GTT GG-3′; TNFα fwd: 5′-AGC CCC CAG TCT GTA TCC TT-3′ rev: 5′-CTC CCT TTG CAG AAC TCA GG-3′; GAPDH fwd: 5′-TGG CAT TGT GGA AGG GCT CAT GAC-3′ rev: 5′-ATG CCA GTG AGC TTG CCG TTC AGC-3′. GAPDH was used as house-keeping gene for normalization.

### Flow Cytometry

Antibodies used for the different staining panels were the following: CD4 (rat anti-mouse, eBioscience); CD25, TCR Vα2, CD8a, B220, CD3, CD4 (rat anti-mouse, BioLegend); CD62L, CD44, CD86, I-A/I-E MHC II, CD40, GR-1, CD11b (rat anti-mouse, BD Biosciences); CD11c, CD86 (hamster anti-mouse, BD Biosciences); CD69 (hamster anti-mouse, BioLegend); CD45.2 (mouse anti-mouse, BD Biosciences). Spleens or lymph nodes (LN) were harvested and passed through a nylon mash to obtain a single cell suspensions. Cells were then stained with the indicated antibodies in FACS buffer (PBS+1% FCS) for 30 min at 4°C. After staining cells were washed and analyzed by flow cytometry in either a FACS-Canto, LSRII or FACS-Verse (BD Biosciences). Data was analyzed using FlowJo.

### Multiphoton Microscopy

Multiphoton imaging was done using a Zeiss LSM7 MP System equipped with a 20x/1.0NA water-immersion objective lens (Zeiss UK, Cambridge, UK) and a tunable Titanium: sapphire solid-state two-photon excitation source (Chamelon Ultra II; Coherent Laser Group, Glasgow, UK) and optical parametric oscillator (OPO; Coherent Laser Group). Popliteal LNs were excised 24 h after immunization, transferred into CO_2_-independent media at room temperature and bound onto a plastic coverslip with veterinary glue (Vetbond, 3M, St. Paul, MN). Grease was used to attach the coverslip to the bottom of the imaging chamber, which was supplied with warmed (36.5°C) and gassed (95% O_2_ and 5% CO_2_) RPMI 1640 before and during the imaging. A laser output of 820 nm and OPO signal at 1,060 nm provided excitation of YFP CD11c- and DS-Red OTII T cells. Acquisition of the videos was done for 20–30 min with X-Y pixel resolution of 512 × 512 inches in 2 μm. Cellular 3D tracking was done using Volocity 6.1.1 (Perkin Elmer, Cambridge, UK). Mean velocity, displacement and meandering index was calculated for each object. Intersection of DsREd and YFP objects was used to determine interaction between T cells and DCs, respectively (28, 30).

### Statistical Analysis

Results are shown as mean ± SEM unless otherwise stated, statistical significance was determined using two-tailed Mann-Whitney-test, when multiple comparisons were made Bonferroni correction was used. A *p*-value < 0.05 was considered significant.

## Results

### Reduced Host Response to VSV Infection in miR-155 Deficient Mice

**Figure 1:**
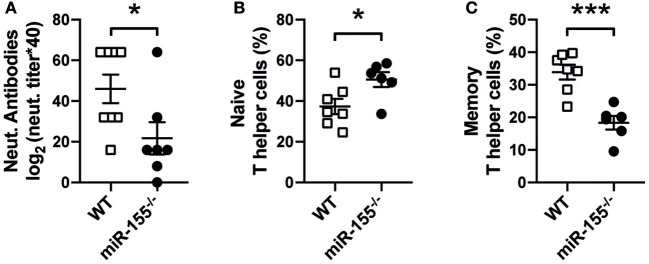
Reduced host response to VSV infection in miR-155 deficient mice. **(A)** MiR-155^−/−^ and WT mice were infected intravenously with Ovalbumin-expressing rVSV (2 × 10^5^ PFU/mL). After 8 days of infection, animals were sacrificed and serum and spleens were collected. Quantification of anti-VSV neutralizing antibodies present in mouse serum was done via VSV-specific plaque assay, as explained in material and methods Frequencies of naïve Th cells (CD62L^+^CD4^+^) **(B)** and memory Th cells (CD44^+^CD4^+^) **(C)** present in the spleen were quantified by flow cytometry. Data is presented as mean ± SEM, and representative of two independent experiments (*n* = 6–8 mice). Statistical significance was calculated using two-tailed Mann-Whitney test. *p ≤ 0.05, ***p < 0.001.

To test whether miR-155 is involved in the generation of anti-viral responses to cytopathic viral infection, wild-type (WT) and miR-155 deficient mice (miR-155^−/−^) were infected with VSV. In serum of infected animals on day 8, miR-155 deficient mice showed a significant decrease in neutralizing antibodies against VSV (Figure 1A). In addition, we detected increased numbers of naïve (CD4^+^CD62L^+^CD44^−^) and reduced numbers of memory (CD4^+^CD62L^−^CD44^+^) T cells in miR-155 deficient mice compared to WT mice (Figures 1B,C), demonstrating both impaired antiviral antibody production as well as decreased T helper cell activation during VSV infection in miR-155 deficient mice.

### DC Maturation Is Not Affected by Absence of miR-155

**Figure 2:**
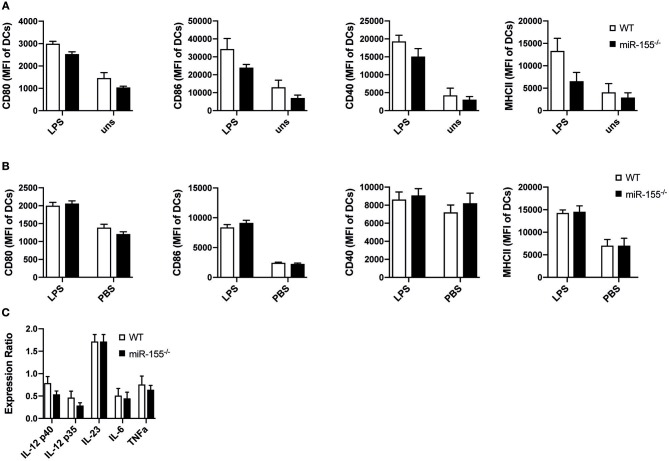
MiR-155 deficiency does not modify activation and function of DCs. **(A)** WT and miR-155^−/−^ BMDCs cultured for 24 h in the presence or absence of LPS (10 μg/mL) were analyzed for the expression of CD80, CD86, CD40, and MHC II (MHC II) by flow cytometry. Bars show mean fluorescence intensity (MFI) ± SEM of CD11c^+^Gr-1^−^ gated cells (*n* = 5 per condition, pooled from two independent experiments). **(B)** MFI ± SEM of the expression of CD80, CD86, CD40, MHC II of splenic CD11c^+^Gr-1^−^ cells of WT and miR-155^−/−^ mice 6 h after intraperitoneal injection of LPS (75 μg/mouse) or PBS (*n* = 5 per condition). **(C)** CD11c^+^Gr-1^−^ cells were FACS sorted from spleens of mice treated with LPS for 6 h and mRNA expressions levels of the pro-inflammatory cytokines IL-12, IL-23, IL-6, and TNFα was quantified by qPCR (*n* = 5). Data is presented as mean ± SEM. Statistical significance was calculated using two-tailed Mann-Whitney test with Bonferroni correction.

We next asked, whether reduced activation of T helper cells could be due to impaired function of antigen presenting cells (APCs). So far, the role of miR-155 in the activation and function of APCs, especially DCs, has been controversial (5, 6). Therefore, we analyzed how miR-155 affects dendritic cell function both *in vitro* and *in vivo* in our setting. First, we generated bone marrow-derived dendritic cells (BMDCs) from WT and miR-155^−/−^ mice and evaluated the expression of co-stimulatory molecules. Upregulation of CD80 (*p* = 0.1268), CD86 (*p* = 0.444), CD40 (*p* = 0.444) as well as MHC II (*p* = 0.444) were not significantly different 24 h post LPS stimulation (Figure 2A).

We next tested maturation of DCs *in vivo* by analyzing up-regulation of maturation markers and the expression of pro-inflammatory cytokines of CD11c^+^ DCs after LPS administration intraperitoneally in WT and miR-155^−/−^ mice. Up-regulation of CD80 (*p* = 1), CD86 (*p* = 0.8344), CD40 (*p* = 1), and MHC II (*p* = 1) was similar and independent of miR-155 presence (Figure 2B). To evaluate mRNA expression levels of pro-inflammatory cytokines, we sorted CD11c^+^ cells and quantified RNA expression levels of IL-12, IL-23p19, IL-6, and TNFα by qPCR. We saw no differences between miR-155^−/−^ and WT CD11c^+^ DCs (Figure 2C). In addition, we quantified the amount of pro-inflammatory cytokines present in the serum after LPS administration and saw that miR-155 deficient mice produced a similar amount of IL-6, TNFα, and IL-12 as compared to WT mice (Figure S1).

### Lack of miR-155 Does Not Influence T Cell Stimulatory Capacity of DCs

**Figure 3:**
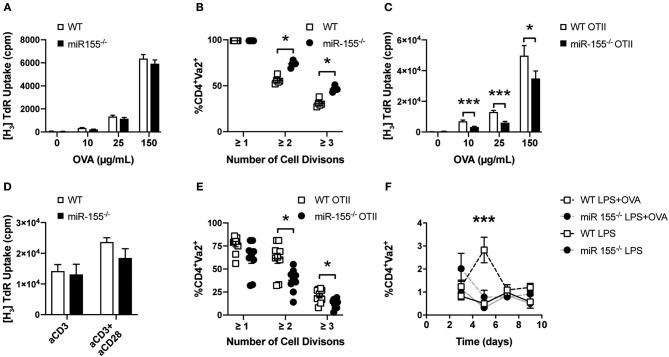
Absence of miR-155 in CD4^+^ T cells leads to reduced clonal expansion. **(A)** WT or miR-155^−/−^ BMDCs were co-cultured with WT OTII T cells in a 1:5 ratio in the presence of the indicated concentrations of OVA. Proliferation was quantified by H_3_-Thymidine incorporation. Results show mean values ± SEM of 6 technical replicates using two mice per genotype. The plot is representative of 3 independent experiments. **(B)** OTII T helper cells were stained with CFSE and transferred *i.v*. into WT and miR-155^−/−^ mice. One day later mice were immunized *i.v*. with LPS (75 μg/mouse) and OVA (30 μg/mouse). Cell proliferation was quantified 3 days after immunization using flow cytometry by measuring CFSE mean fluorescence intensity of Vα2^+^ cells, gated on CD4^+^ cells in the spleen (*n* = 5 for each genotype). **(C)** WT or miR-155^−/−^ OTII T cells were co-cultured with WT BMDCs in a 1:5 ratio in the presence of the indicated concentrations of OVA. Proliferation was quantified by H_3_-Thymidine incorporation. Results show mean values ± SEM of 6 technical replicates and two mice per genotype. The plot is representative of 3 independent experiments. **(D)** CD4^+^ cells were MACS isolated from spleen of WT or miR-155^−/−^ mice and seeded in 96 well plates pre-coated with anti-CD3 (1 μg/mL) and anti-CD28 (3 μg/mL). Proliferation was quantified by H_3_-Thymidine incorporation. Results show mean values ± SEM of 6 technical replicates and two mice per genotype. The plot is representative of 3 independent experiments. **(E)** WT or miR-155^−/−^ OTII T cells were stained with CFSE and transferred *i.v*. into WT. Mice were immunized *i.v*. with LPS (75 μg/mouse) with OVA (30 μg/mouse). Cell expansion was quantified 3 days after immunization using flow cytometry by measuring CFSE mean fluorescence intensity of Vα2^+^ cells, gated on CD4 ^+^ cells (*n* = 8 for each genotype). **(F)** WT or miR-155^−/−^ OTII T helper cells were transferred *i.v*. into CD45.1 mice (immunization was done as referred above). Frequency of CD45.2^+^Vα2^+^ cells of gated CD4 ^+^ cells, present 3, 5, 7, or 9 days after immunization was done using flow cytometry (*n* = 3 per condition and genotype). Data is presented as mean ± SEM, statistical significance was calculated using two-tailed Mann-Whitney test with Bonferroni correction for all panels except **(F)** where Two-way ANOVA was used. Significance shown reflect comparison of WT vs. miR-155^−/−^. *p < 0.05, ***p < 0.001.

Next, we were interested in determining whether the absence of miR-155 in dendritic cells affected their capacity to activate CD4^+^ T cells. To this end, we loaded WT or miR-155^−/−^ BMDCs with ovalbumin (OVA) and co-cultured them with OTII T cells expressing a transgenic T cell receptor (TCR) recognizing the OVA peptide 323–339. We observed that the proliferation of OTII T cells induced by miR-155 deficient or sufficient BMDCs was similar, regardless of the concentration of OVA (Figure 3A). To analyze the impact of miR-155 on the capacity of APCs to stimulate T cells *in vivo*, we transferred CFSE labeled OTII T cells into WT and miR-155^−/−^ mice and quantified their proliferation after immunization with OVA and LPS by CFSE dilution. Proliferation (i.e., CFSE dilution) of WT OTII T cells was even increased when these cells were transferred to miR-155 deficient mice compared to WT mice (Figure 3B). Taken together, our data demonstrates that the absence of miR-155 does not affect phenotypic maturation and MHC class II presentation capacities of DCs neither *in vitro* nor *in vivo*.

### Lack of miR-155 Affects the Proliferative Capacity of CD4^+^ T Cells

To investigate the role of miR-155 in T helper cell activation and proliferation, we crossed miR-155^−/−^ mice with OTII mice to generate miR-155 sufficient (WT OTII) and deficient (miR-155^−/−^ OTII) OTII mice. We then co-cultured WT OTII and miR-155 OTII CD4^+^ T cells with WT BMDCs in the presence of different OVA concentrations and quantified their proliferation. As shown in Figure 3C, miR-155^−/−^ OTII T cells proliferated significantly less than their WT counterparts after stimulation. When we measured levels of different cytokines present in the supernatants of these co-cultures, we also found a significantly reduced production of IL-2 and IFN-γ (Figure S2). However, when we used plate bound anti-CD3 or anti-CD3/CD28 to stimulate either MACS isolated or FACS-sorted CD4^+^ T cells, there were no significant differences in the proliferative response (Figure 3D) or in cytokine production (Figure S3) between WT and miR-155 deficient cells.

We then tested whether miR-155 expression affected CD4^+^ T cell proliferation *in vivo*. For this, we transferred CFSE labeled WT OTII or miR-155^−/−^ OTII T cells into WT mice and immunized these with LPS alone or the combination of LPS and OVA. In order to easily identify the transferred OTII T cells (derived from CD45.2 mice), we used CD45.1 mice as recipients. In line with our *in vitro* data, there was a lower proliferative response of the transferred miR-155^−/−^ OTII T cells in comparison to WT OTII T cells *in vivo* (Figure 3E). We also measured the relative abundance of transferred OTII T cells over time. Mice received WT or miR-155^−/−^ OTII T cells with or without immunization with LPS-OVA. In non-immunized mice, the relative numbers of transferred OTII T cells remained constant at all time points analyzed. In mice immunized with LPS+OVA that had received WT OTII T cells, there was a significant expansion of the transferred OTII T cells at day 5 after immunization. In contrast we did not detect increased numbers of transferred miR-155^−/−^ OTII T cells after immunization at all time points analyzed (Figure 3F). These data suggest that miR-155 is necessary for proliferation and expansion after immunization of CD4^+^ T cells after immunization *in vivo*.

### CD4^+^ T Cell Interaction With DCs Is Controlled by miR-155

**Figure 4:**
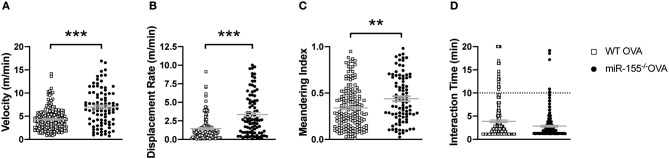
MiR-155 in CD4^+^ T cells is required for proper DC-T cell interactions. CD11c-YFP mice received 5 × 10^6^ WT or miR-155 OTII T cells labeled with CMTPX and were immediately immunized with OVA/CFA in the footpad. Popliteal LNs were collected 24 h after immunization, three random regions of each LN were imaged and Volocity software was used for semi-automated tracking. Each point represents a single tracked object, either a single cell or the interaction (intersection between the DC and T cell fluorescent signal). Mean velocity **(A)**, displacement rate **(B)**, meandering index **(C)**, and interaction time between DCs and T cells **(D)** were quantified using Volocity software programs. Data are presented as mean ± SEM, two-tailed Mann-Whiney test and representative of two animals per group of at least two independent experiments. ***p ≤ 0.001, **p ≤ 0.01.

As our previous experiments demonstrated a reduced activation of miR-155 deficient CD4^+^ T cells only upon activation by APCs, which could be overcome by antibody-mediated direct stimulation of CD3 and CD28, we wanted to investigate the mechanism leading to this striking difference in more detail. It has been shown that the quality of DC and CD4^+^ T cell interaction is crucial for efficient T helper cell response (30, 31). Using multiphoton microscopy, we investigated whether lack of miR-155 in T cells could affect DC/T cell interaction. To this end, miR-155 sufficient or deficient OTII T cells were labeled with the fluorescent dye CMTPX and then transferred into WT CD11c-YFP mice that were immunized with OVA in CFA on the same day of cell transfer. After 24 h, popliteal lymph nodes (LN) were collected and imaged. Analysis of three different areas in each LN revealed that miR-155^−/−^ OTII T cells moved further (*p* = 0.0001), with a higher velocity (*p* = 0.0001) and with a higher meandering index (*p* = 0.0018) compared to WT OTII (Figures 4A–C; Videos S1, S2). Additionally, we analyzed the formation of clusters in the LN and their interaction times. There were numerically more stable clusters of WT OTII T cells with CD11c^+^ cells than of miR-155^−/−^ OTII T cells (Figure 4D, *p* = 0.6290). These data suggest, that miR-155 deficiency in CD4^+^ T cells is crucial for correct DC/T cell interaction.

### miR-155 Deficiency in T Helper Cells Leads to Reduced B Cell Expansion and Antibody Production

**Figure 5:**
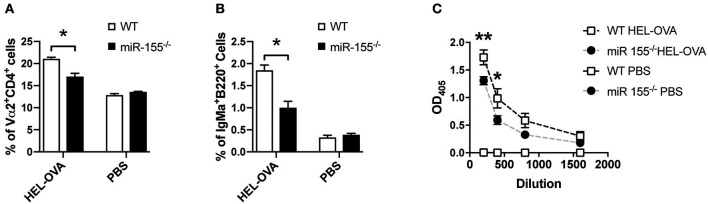
MiR-155 deficiency in T cells affects T cell help to B cells. **(A)** WT mice received 5 × 10^6^ HEL-specific B cells and 5 × 10^6^ WT or miR-155 OTII T cells and brachial LN were collected 6 days after immunization with HEL-OVA/CFA. Expansion of transferred OTII T cells was quantified by flow cytometry as frequency of Vα2^+^ cells gated on CD4^+^ cells. **(B)** Expansion of HEL-specific B cells was also analyzed by quantifying IgMa^+^ cells, gates on B220^+^ cells. **(C)** Serum was collected at experimental endpoint and assessed for the presence of anti-HEL IgMa by ELISA. Data are presented as mean ± SEM and representative of two different experiments (*n* = 6). Statistical significance was calculated using two-tailed Mann-Whitney test with Bonferroni correction in **(A,B)**, while Two-way ANOVA was used in **(C)**. *p ≤ 0.05, **p ≤ 0.01.

Having established that miR-155 in CD4^+^ T cells is important for APC-mediated proliferation and cytokine production *in vitro* and *in vivo*, we asked, whether this is relevant during viral infection. Using an ovalbumin encoding transgenic VSV (VSV-Ova), we first analyzed, whether infusion of WT OTII T cells could boost the generation of neutralizing antibodies. However, administration of neither WT nor miR-155 deficient OTII T cells changed the levels of neutralizing antibodies (Figure S4). To test, whether miR-155 plays a role in the B cell stimulatory capacity of T cells in a different setting, we transferred miR-155 sufficient and deficient OTII T cells and B cells of BCR transgenic mice, which express a BCR recognizing HEL. This system allows evaluation of anti-HEL B cell responses which depend on help from OTII T cells in response to a HEL-OVA fusion protein (28). Immunization with HEL-OVA+CFA led to an expansion of WT OTII T cells; in contrast miR-155^−/−^ OTII T cells expansion was significantly lower (Figure 5A, *p* = 0.0316). When we analyzed the capacity of OTII T cells to induce expansion of antigen specific B cells, we found significantly reduced HEL BCR transgenic B cells in mice that had received miR-155^−/−^ OTII T cells compared to WT OTII T cells (Figure 5B, *p* = 0.0316). The production of anti-HEL IgMa production was also affected by the lack of miR-155 on the T cells, since there were significantly less anti-HEL IgMa antibodies in the serum of animals which received miR-155 deficient OTII T cells (Figure 5C, 200 dilution *p* = 0.0060, 400 dilution *p* = 0.0122, 800 dilution *p* = 0.2385, 1,600 dilution *p* = 1). This data clearly demonstrates an important role for miR-155 in the activation of B cells by T helper cells.

### Absence of miR-155 Impacts CD4^+^ T Cell Response to rVSV and Vaccinia Virus

**Figure 6:**
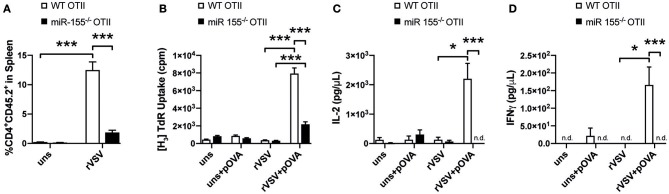
MiR-155 deficiency diminishes T helper cell activation during viral infection. **(A)** CD45.1 mice received 3 × 10^6^ WT or miR-155 or WT OTII T cells, mice were infected *i.v*. with rVSV the following day. Spleens were collected 8 days after infection. Expansion of transferred cells was quantified as percentage of CD45.2^+^ from all CD4^+^ cells. **(B)** Splenocytes were cultured for 3 days in full RPMI with or without OVA peptide (323–339) and proliferation was quantified by H_3_-Thymidine incorporation. Flow Cytomix was used to quantify levels of IL-2 **(C)** and IFN-γ **(D)** present in the supernatant of splenocyte re-stimulation cultures. Data is presented as mean ± SEM and representative of two independent experiments (*n* = 6). Statistical significance was calculated using two-tailed Mann-Whitney test with Bonferroni correction. *p ≤ 0.05; ***p ≤ 0.001.

Previous studies showed a role of miR-155 in the CD8^+^ T cell response to viral infection (18, 19, 32). However, also CD4^+^ T cells are important in anti-viral defense (23, 33) and therefore we were interested whether miR-155 was involved in the CD4^+^ T cell mediated response to viral infection. To study this, we adoptively transferred miR-155^−/−^ and WT OTII T cells isolated from CD45.2 mice into CD45.1 WT recipient mice and infected these with rVSV-Ova. Eight days after infection, 12% of the total CD4^+^ T cells in the spleen were CD45.2^+^, suggesting a robust expansion of WT OTII T cells after viral infection. In contrast, in mice that had received miR-155 deficient OTII T cells before infection with rVSV-Ova, only 2% of the overall CD4^+^ population were CD45.2^+^ on day 8 (Figure 6A). To analyze the recall response of WT and miR-155 deficient OTII T cells *ex vivo*, we harvested the spleens 8 days after rVSV-Ova infection and re-stimulated splenocytes *ex vivo* with OVA 323-339 peptide. This peptide is only recognized by OTII T cells when presented on MHC class II molecules, excluding stimulation of MHC I restricted CD8^+^ T cells. Quantification of proliferation demonstrated that splenocytes from mice which received miR-155 deficient OTII T cells proliferated significantly less than those that had received WT OTII T cells (Figure 6B). In addition, we analyzed cytokine production from re-stimulated splenocytes in the supernatant. We found that supernatants from mice that had received miR-155 deficient OTII T cells contained significantly lower levels of IL-2 (Figure 6C) and IFN-γ (Figure 6D). These data suggest, that miR-155 is necessary for the generation and activation of virus specific CD4^+^ T cells in response to a viral infection.

## Discussion

MicroRNAs have been demonstrated to control various aspects of both innate and adaptive immune responses, both in infections and autoimmunity (34–37). Among them, miR-155 has been shown to be important for the generation of both humoral and cellular immune responses both during infection and autoimmunity (4, 5, 13, 14). MiR-155 has been shown to be critically important during infection with bacteria, but also during viral infection requiring the generation of antiviral CD8^+^ T cells. In this study, we demonstrate that miR-155 is required for the efficient generation of CD4^+^ T cell and humoral responses to the cytopathic virus VSV. We show that lack of miR-155 severely impairs efficient interaction of T cells with APCs, inhibiting subsequent activation and cytokine production of virus specific T cells in a T cell intrinsic fashion.

While the role of T helper cells in viral infection is not completely understood, the ability of CD4^+^ T cell to produce cytokines has been linked to protection from lethal infection with VSV. In addition, T helper cell-derived IL-2 has been demonstrated to be crucial for the generation of anti-viral CD8^+^ T cells in VSV infection (23–25). In our experiments, using transgenic ovalbumin expressing VSV, we demonstrate that without miR-155, T helper cells fail to expand during viral infection and are incapable of producing significant amounts of IL-2 and IFN-γ. Our *in vivo* immunization experiments with ovalbumin and additional *in vitro* experiments demonstrate that this failure in activation is T cell intrinsic, as miR-155 deficient APCs are fully capable of activating WT OTII T cells *in vitro*. In line, while we did notice numerically reduced expression of co-stimulatory molecules of BMDCs derived from miR-155 deficient mice *in vitro*, we did not detect differences of DC maturation upon TLR4 stimulation *in vivo* in the absence of miR-155. In addition, proliferation of WT OTII T cells transferred into miR-155 deficient hosts was even enhanced compared to proliferation of WT OTII T cells transferred into WT hosts, demonstrating that in our system miR-155 deficiency in APCs does not inhibit their ability to stimulate T cell proliferation. Of note, and in line with our data, Rothchild et al. also noted increased capacities of miR-155 deficient hosts to activate CD4^+^ T cells during mycobacterial infection (38).

We also detected decreased titers of neutralizing antibodies against VSV in miR-155 deficient mice. While the role of miR-155 in B cells has been analyzed extensively, and plays an important role in immunoglobulin class switch (8, 9), recent reports have also identified miR-155 as an important regulator of follicular T helper cell development (12, 39). In our experiments, employing a HEL-OVA fusion protein, we could demonstrate an important role of miR-155 in T cells and in their capacity to induce antigen specific B cell proliferation and antibody production. Our results suggest that the reduced anti-viral titers might be a consequence of insufficient T cell help.

Mechanistically, we could show that miR-155 deficient T cells form complexes with antigen bearing APCs after immunization *in vivo* that are of reduced stability compared to the complexes formed between APCs and WT T cells, suggesting impaired APC-T cell communication in the absence of miR-155 in T cells. In line with this assumption, there was no difference in the ability of WT or miR-155 deficient T helper cell to proliferate in response to anti-CD3 and/or anti-CD28 stimulation *in vitro*, a fact that has been noted by others as well (12). However, in all *in vitro* and *in vivo* assays that required APC-T cell interaction there was reduced a proliferation of CD4^+^ T cells lacking miR-155 compared to their WT counterparts. The most dramatic effects were observed in our viral infection models, were we could hardly detect any activation/proliferation in the ovalbumin specific (i.e., virus specific) miR-155 deficient T cells. It will be interesting to determine the factors responsible for this effect. Of note, miR-155 has been shown to control leukocyte adhesion to brain endothelium (40), rendering adhesion molecules regulated by miR-155 possible mediators of the observed impaired APC-T cell communication.

Taken together, these data suggest that during viral infection, proper APC-T cell interactions are crucial for the generation of virus specific CD4^+^ T cell responses and that altering this communication has dramatic consequences. It would be interesting to investigate, whether APC-T cell communication is also perturbed in CD8^+^ T cell activation.

## Data Availability

All datasets generated for this study are included in the manuscript and/or the Supplementary Files.

## Ethics Statement

This study was carried out in accordance with the recommendations of animal ethics committee of the Medical University Vienna and of the University of Glasgow. The protocol was approved by the animal ethics committee of the Medical University Vienna and of the University of Glasgow.

## Author Contributions

EG-A, CS, MK-S, RB, and SB designed research. EG-A, VS, CS, RB, MK-S, JSB, AP, and BP performed research. EG-A, VS, CS, RB, MK-S, JSB, AP, BP, KR, MB, JB, AB, and GS interpreted the data. AB, RB, MK-S, and JB contributed vital reagents. EG-A, VS, CS, JSB, JS, KR, MB, JB, AB, GS, and SB wrote the paper. All authors critically revised and approved the manuscript and are accountable for the accuracy and integrity of the work.

### Conflict of Interest Statement

The authors declare that the research was conducted in the absence of any commercial or financial relationships that could be construed as a potential conflict of interest.

## Supplementary Material

Figure S1 WT and miR-155 mice were injected with LPS i.p. After 6 hours, serum was collected and the presence of IL-6 **(A)**, TNFα **(B)** and IL-12p40 **(C)** was quantified using Cytoplex analysis (*n* = 4). Data is presented as mean ± SEM.

Figure S2 WT or miR-155^−/−^ OTII T cells were co-cultured with WT BMDCs. Supernatants were analyzed for the presence of **(A)** IL-2 and **(B)** IFN-γ. ^***^*p* < 0.001. Data was pooled from three different experiments each with 2 animals per genotype. Data is presented as mean ± SEM, statistical significance was calculated using two-tailed Mann-Whitney test with Bonferroni correction.

Figure S3 CD4^+^ cells were isolated from spleen of WT or miR-155^−/−^ mice and seeded in 96 well plates pre-coated with anti-CD3 or anti-CD3 and anti-CD28. Supernatants were analyzed for the presence of **(A)** IL-2, **(B)** IFN-γ, **(C)** TNF, **(D)** IL-4. Data was pooled from three different experiments each with 2 animals per genotype. Statistical significance was calculated using two-tailed Mann-Whitney test with Bonferroni correction.

Figure S4 CD45.1 mice received 3x106 miR-155^−/−^ or WT OTII T cells were infected 24 hours later with rVSV-OVA. Serum was collected 8 days after infection and rVSV-OVA neurtralyzing antibodies were quantified by plaque assay as explained in methods section. Results represented as mean ± SEM of two different experiments with six animals per group.

Video S1 19-min capture of popliteal LNs excised from CD11c YFP C57BL/6 mice that received WT OTII T cells and were immunized in the footpad with CFA/OVA. Red cells represent OTII T cells transferred (CMTPX) and green are DCs (YFP) present in the LN.

Video S2 19-min capture of popliteal LNs excised from CD11c YFP C57BL/6 mice that received miR155^−/−^ OTII T cells and were immunized in the footpad with CFA/OVA. Red cells represent OTII T cells transferred (CMTPX) and green are DCs (YFP) present in the LN.
