# Supplementary figures and images for: MicroRNA-155 Controls T Helper Cell Activation During Viral Infection
Source: Front Immunol. 2019 Jun 13;10:1367. doi: 10.3389/fimmu.2019.01367 (PMC6593301; doi:10.3389/fimmu.2019.01367)

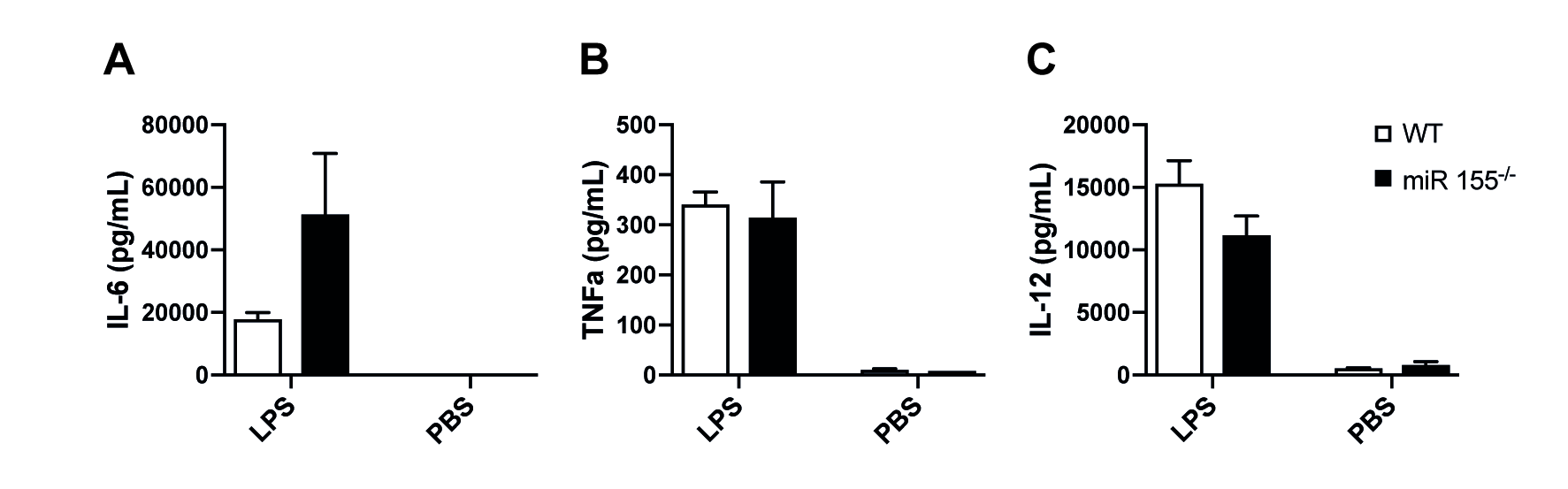

Supplement: Figure S1 — WT and miR-155 mice were injected with LPS i.p. After 6 hours, serum was collected and the presence of IL-6 (A), TNFα (B) and IL-12p40 (C) was quantified using Cytoplex analysis (n = 4). Data is presented as mean ± SEM. [file Image_1.TIF]

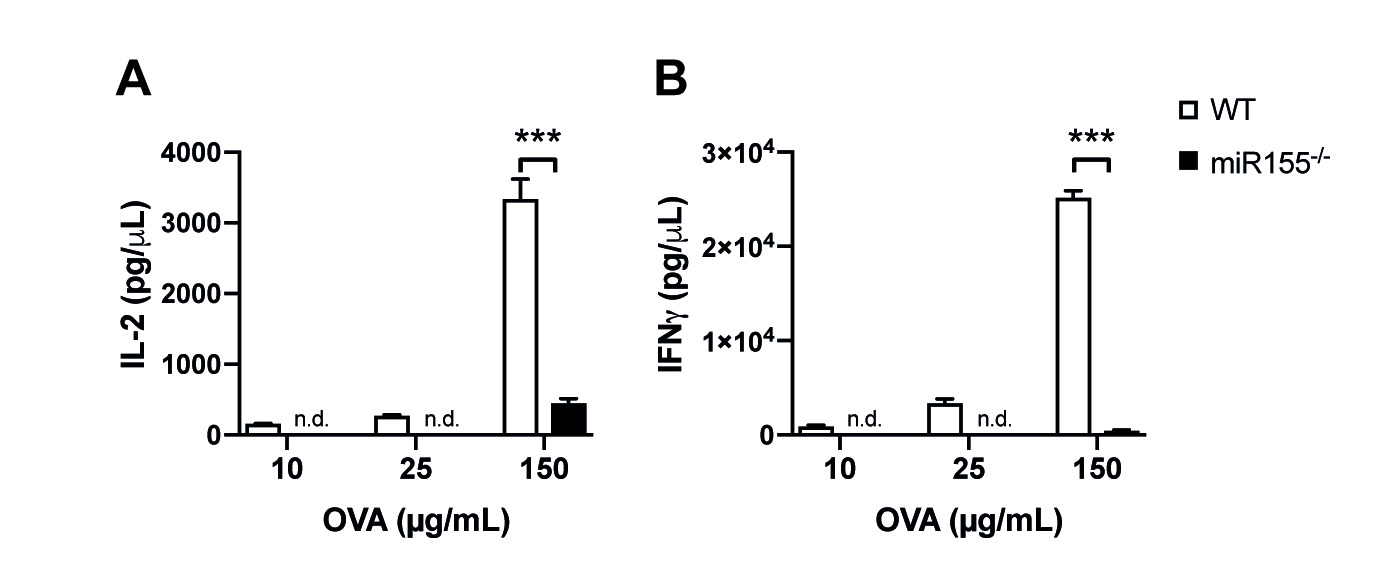

Supplement: Figure S2 — WT or miR-155−/− OTII T cells were co-cultured with WT BMDCs. Supernatants were analyzed for the presence of (A) IL-2 and (B) IFN-γ. ***p < 0.001. Data was pooled from three different experiments each with 2 animals per genotype. Data is presented as mean ± SEM, statistical significance was calculated using two-tailed Mann-Whitney test with Bonferroni correction. [file Image_2.TIF]

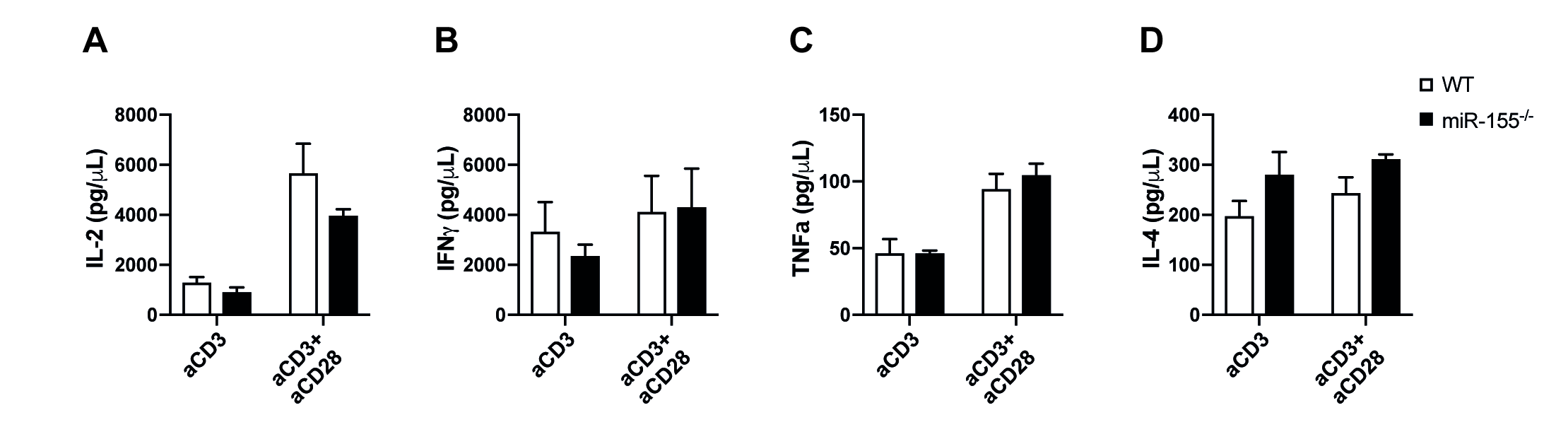

Supplement: Figure S3 — CD4+ cells were isolated from spleen of WT or miR-155−/− mice and seeded in 96 well plates pre-coated with anti-CD3 or anti-CD3 and anti-CD28. Supernatants were analyzed for the presence of (A) IL-2, (B) IFN-γ, (C) TNF, (D) IL-4. Data was pooled from three different experiments each with 2 animals per genotype. Statistical significance was calculated using two-tailed Mann-Whitney test with Bonferroni correction. [file Image_3.TIF]

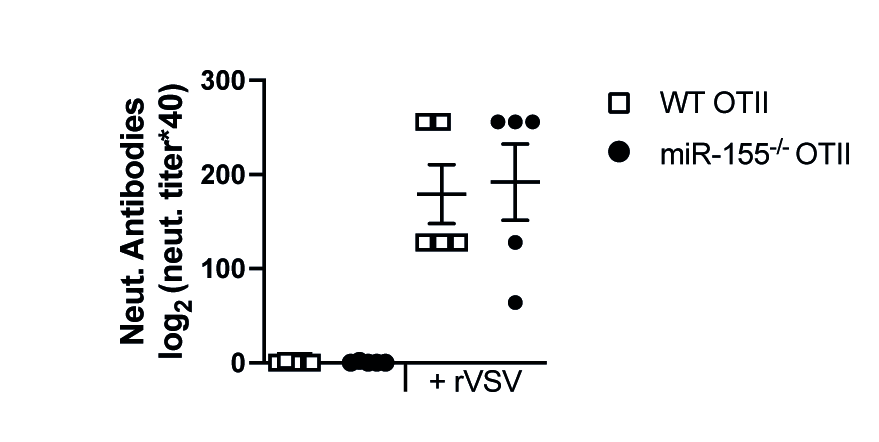

Supplement: Figure S4 — CD45.1 mice received 3x10^6^ miR-155−/− or WT OTII T cells were infected 24 hours later with rVSV-OVA. Serum was collected 8 days after infection and rVSV-OVA neurtralyzing antibodies were quantified by plaque assay as explained in methods section. Results represented as mean ± SEM of two different experiments with six animals per group. [file Image_4.TIF]
